# Balloon Rupture-Facilitated Grenadoplasty in Complex Left Anterior Descending Chronic Total Occlusion

**DOI:** 10.1016/j.jaccas.2026.107408

**Published:** 2026-03-25

**Authors:** Pierluigi Demola, David Rutigliano, Sergio Rutigliano, Pierangelo Basso, Francesco Cassano, Vincenzo Massimo Bonfantino

**Affiliations:** a Department of Cardiology, Ospedale della Murgia “F. Perinei”, ASL Bari, Bari, Italy; b Cardiology Department, San Paolo Hospital, Bari, Italy; c UOC di Cardiologia—UTIC, Ospedale “Di Venere”, ASL Bari, Bari, Italy

**Keywords:** complication, coronary circulation, percutaneous coronary intervention

## Abstract

**Background:**

Percutaneous coronary intervention (PCI) for chronic total occlusion (CTO) is indicated in selected patients with symptoms or significant ischemic burden, balancing clinical benefit against procedural risk.

**Case Summary:**

A 66-year-old man with inferolateral ST-segment elevation myocardial infarction was found to have a calcified ostial left anterior descending artery CTO. During staged antegrade CTO PCI, high-pressure balloon inflation at the proximal cap resulted in an unintentional balloon rupture, creating a focal microdissection without perforation. This event enabled true-lumen wiring, lesion preparation, and successful stent implantation with optimal angiographic and intravascular ultrasound results.

**Discussion:**

The mechanism was consistent with balloon-assisted microdissection rather than hydrodynamic contrast recanalization. Although unintentional, the rupture facilitated plaque modification and re-entry.

**Take-Home Message:**

Unintentional balloon rupture may rarely act as a bailout mechanism during complex CTO PCI but should never be considered a routine strategy.

## Visual Summary

**Figure:**
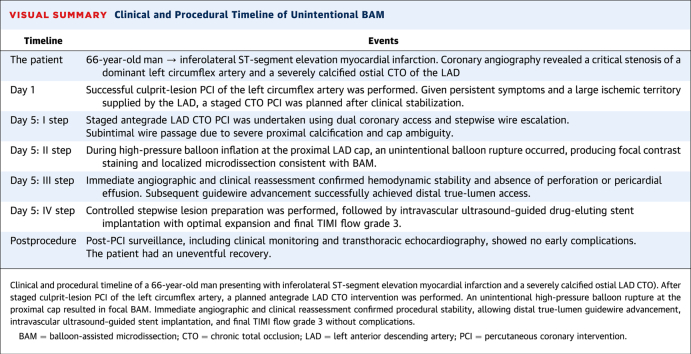

Percutaneous coronary intervention (PCI) for chronic total occlusion (CTO) remains one of the most technically demanding procedures in contemporary interventional cardiology.^1^ In carefully selected patients with symptoms or a significant ischemic burden, CTO PCI may provide clinical benefit, but it requires meticulous procedural planning and stepwise escalation strategies, particularly in the presence of severe calcification.

Balloon-assisted microdissection (BAM), also referred to as “grenadoplasty,” has been described as a bailout technique to facilitate lesion modification in balloon-uncrossable or undilatable calcified segments.^2^ However, balloon rupture carries an inherent risk of vessel injury and should not be considered a routine strategy.

We report a case of complex left anterior descending artery (LAD) CTO PCI in which an unintentional high-pressure balloon rupture created a focal microdissection, unexpectedly facilitating true-lumen crossing and successful revascularization. This case highlights the underlying mechanism, safety considerations, and practical lessons of BAM when encountered as an inadvertent bailout event.

## Case Presentation

A 66-year-old man with several risk factors was referred to our emergency department for cardiac chest pain, revealing an ST-segment elevation in the inferolateral leads.

The invasive coronary angiography revealed a 99% stenosis of the II segment of a dominant left circumflex artery (LCx) and a calcific ostial CTO of the LAD; the coronary anatomy shows a separate origin of the LAD from the LCx, with no presence of left main.

Given persistent angina and a relevant ischemic territory supplied by the LAD, a staged CTO PCI was planned after discussion of expected benefit and procedural risk, and the Japanese chronic total occlusion score was 3. A 6-F extra backup guiding catheter and an 8-F extra backup guiding catheter were used to cannulate both LCx and LAD separately. A Turnpike LP microcatheter (Teleflex) was used for attempting wire crossing of the LAD by an XT-A guidewire (Asahi Intecc).

**Figure 1:**
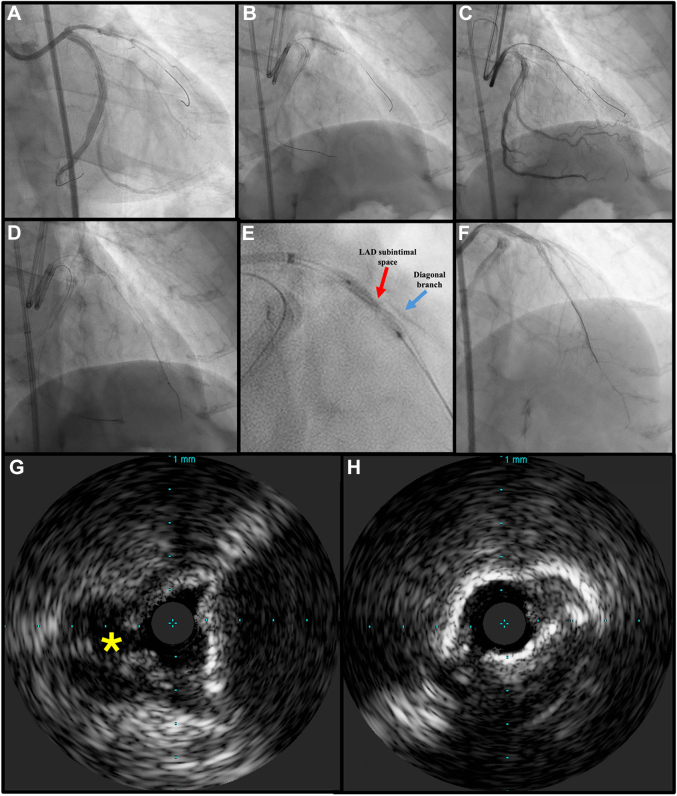
Procedural Steps of Unintentional Balloon-Assisted Microdissection During LAD CTO PCI (A) Baseline angiography showing calcified ostial chronic total occlusion of the proximal left anterior descending artery (LAD); (B) accidental balloon rupture producing localized contrast staining and focal microdissection by a “grenade-like” appearance in LAD architecture; (C) XT-A guidewire in the architecture of the II segment of the LAD, confirmed by angiographic control; (D) distal injection by a microcatheter, confirming the gain of the true lumen of the LAD; (E) angiographic evidence of a balloon “dog-boning effect” within the proximal LAD subintimal space (red arrow), confirming the presence of a circumferential calcific lesion. The proximal diagonal branch is faintly visualized adjacent to the proximal cap (blue arrow). (F) Final angiographic result; (G) intravascular ultrasound (IVUS) at the entry site of the diagonal (yellow asterisk, ∗); (H) IVUS calcific cap, slightly after bifurcation with the diagonal branch in the LAD.

The antegrade wire escalation technique with the GAIA II guidewire (Asahi Intecc) enabled the first diagonal branch crossing, which was also compromised by a high calcific burden. Then, using a dual-lumen microcatheter and an XT-A, we attempt to access the LAD lumen. The XT-A was always revealed to be in the subintimal space, so we upgraded to a GAIA II guidewire, even though we were not able to gain the true lumen. At this point, we attempt to modify the entry point by inflating a 2.00 semicompliant balloon located at this bifurcation (Figure 1A and Video 1). At 16 atm, the balloon ruptured unexpectedly, producing localized contrast staining and a brief “grenade-like” appearance inside LAD architecture (Figure 1B).

The patient remained hemodynamically stable, with no angiographic evidence of perforation or pericardial effusion. Immediate postprocedural checks, including careful angiographic reassessment and clinical monitoring, confirmed vessel integrity.

At this point, an XT-A easily engaged the true lumen of the LAD, confirmed by angiographic control (Figure 1C). Interestingly, this involuntary “side-granedoplasty” by unintended balloon rupture created a focal microdissection plane (Video 2, Video 3, Video 4).

This event enabled the passage of the XT-A and Turnpike microcatheter true-to-true to the distal LAD, confirmed by distal injection puff (Figure 1D), allowing a subsequent progressive balloon-based dilation (Figure 1E) and drug-eluting stent implantation.

The final angiogram demonstrated excellent 3.00 × 34 mm zotarolimus-eluting stent apposition and TIMI flow grade 3 (Figure 1E), proximal expanded till 3.50 mm. Intracoronary imaging by intravascular ultrasound shows the proximal calcific cap of the LAD in Figure 1F, next to the D1 ostium, and the cracked calcific “napkin ring” right before the distal CTO cap of the LAD in Figure 1G.

Postprocedural patient surveillance included continuous hemodynamic monitoring and transthoracic echocardiography, which excluded pericardial effusion or other complications.

This event reproduced the mechanical principle of BAM, also referred to as “grenadoplasty,” a bailout mechanism in which balloon rupture can facilitate focal plaque modification in balloon-uncrossable or undilatable lesions. In the present case, the rupture occurred unintentionally during high-pressure balloon inflation, transforming a potential complication into a procedural advantage by enabling true-lumen crossing. Importantly, this phenomenon should not be considered a routine or first-line strategy. Intentional balloon rupture, if ever contemplated, should be reserved only as a last-resort bailout in highly selected cases, given the substantial risk of vessel injury and perforation, particularly within calcified subintimal planes. However, in the present case, the phenomenon occurred serendipitously, transforming a potential complication into a procedural advantage.

Originally described as a rescue method to overcome device uncrossability in calcified or fibrotic lesions,^2^ BAM can create focal microdissections that improve device delivery without specialized atherectomy tools.^3^ Subsequent experience has shown that, when cautiously performed, this approach may enable adequate lesion preparation, although uncontrolled dissection or perforation remains possible. This case illustrates how even an unintended balloon rupture can transiently reproduce a similar controlled-fracture mechanism, facilitating subsequent II-level calcium modification balloons or debulking application and optimal stent expansion.^4^^,^^5^

From a practical standpoint, this case underscores several procedural considerations. In heavily calcified CTOs, repeated subintimal wiring despite appropriate antegrade escalation should prompt reassessment and consideration of alternative calcium-modification strategies rather than further forceful escalation. After inadvertent balloon rupture, immediate angiographic and clinical reassessment is essential to exclude perforation and confirm vessel integrity. When distal true-lumen access is achieved under controlled conditions, cautious stepwise lesion preparation and imaging-guided stent optimization may allow safe procedural completion.

Although BAM can be intentionally performed in selected bailout scenarios, this case highlights a crucial procedural insight of an unintentional balloon rupture. When vessel integrity is preserved, and hemodynamics remain stable, such events may unexpectedly reproduce the therapeutic benefit, the safety, and reproducibility of controlled microdissection.

## Funding Support and Author Disclosures

The authors have reported that they have no relationships relevant to the contents of this paper to disclose.

**Take-Home Messages**

• In selected complex calcified chronic total occlusions, unintentional balloon rupture may create a focal microdissection that improves lesion compliance and facilitates true-lumen progression.
• This phenomenon should be considered a bailout occurrence rather than a planned strategy and requires careful procedural reassessment.

## Appendix

Video 1 Short Cineangiogram With Balloon Rupture and Contrast Staining

Video 2 Short Cine Angiogram With Contrast Passage in the True Left Anterior Descending Artery After Balloon-Assisted Microdissection

Video 3 Short Cine Angiogram of Subsequent Left Anterior Descending Artery True-Lumen Wiring

Video 4 Short Cine Angiogram Showing the Final Angiographic Result
